# Effect of Peer Victimization on the Long-Term Mental Health Status among Adults Users of Intellectual Disability Services: A Longitudinal Follow-Up Study

**DOI:** 10.3390/ijerph19074196

**Published:** 2022-04-01

**Authors:** Dai-Rong Yang, Nian-Sheng Tzeng, Fu-Gong Lin

**Affiliations:** 1 National Defense Medical Center, Graduate Institute of Life Sciences, Taipei City 114, Taiwan; 806302038@mail.ndmctsgh.edu.tw; 2 National Defense Medical Center, Department of Psychiatry, Tri-Service General Hospital, School of Medicine, Taipei City 114, Taiwan; pierrens@mail.ndmctsgh.edu.tw; 3 Student Counseling Center, National Defense Medical Center, Taipei City 114, Taiwan; 4 National Defense Medical Center, School of Public Health, Taipei City 114, Taiwan; 5 Department of Optometry, University of Kang Ning, Taipei City 114, Taiwan

**Keywords:** period prevalence, Psychopathology Inventory for Mentally Retarded Adults (PIMRA), psychiatric symptoms, polyvictimization

## Abstract

Caregiving for mental health among people with intellectual disabilities (IDs) in the ID services was reported as insufficient. The purposes of this study were to investigate five types of peer victimization (PV) experiences among adults with ID using ID services, and to gain a deeper understanding of the influence of PV experience on adults with ID’s long-term mental health status. A one-year longitudinal follow-up study was conducted from eight long-term care ID services (*n* = 176). Logistic regression analysis was applied to variables comprising personal characteristics, various types of PV experience and polyvictimization to predict period prevalence of psychiatric symptoms. The data indicated that nearly one-third of individuals with ID experienced at least one psychiatric symptom. The three most common psychiatric symptoms prevalent after one year were adjustment disorder, anxiety disorder, and somatoform disorder. Over the 1-year study period, approximately 40% of adults with ID reported experiencing PV. The most frequently reported types of PV were physical force (26%) and verbal victimization (22%). Polyvictimization was experienced by approximately a quarter of adults with ID. The findings suggest that PV is a common experience among adults in ID services. Thus, for a clearer understanding of mental health risks, caregivers should pay attention to adults with ID who experienced PV.

## 1. Introduction

The prevalence of intellectual disabilities (IDs) worldwide is estimated to be between 1% and 3% [1,2]. In Taiwan, 102,127 people were registered as having ID at the end of 2019, accounting for 0.43% of the country’s population [3]. The government’s welfare expenditure for disabled individuals—those with IDs comprise approximately one-tenth of this group—was USD 784 million in 2006 [4]. In the United States, whose population is 10 times larger than Taiwan’s, the lifetime cost for individuals with ID was estimated to be USD 50 billion in 2000 [5]. In 2019, two of the ten leading causes of disability-adjusted life years in individuals aged 25–49 years were mental disorders [6].

Individuals with IDs exhibit neurodevelopmental deficits characterized by restricted intellectual and adaptive functioning [7]. However, in this population, individuals with mental illnesses are often undiagnosed; difficult to diagnose; or given delayed, inadequate, or no treatment [8,9,10]. Studies have reported that the prevalence of mental illness among individuals with IDs (29–64%) is 3 to 4 times higher than that of individuals without ID (4–26%) [11,12,13]. Thus, diagnosing mental illnesses and promoting mental health among individuals with ID merit greater attention.

Individuals with ID are more likely to experience victimization than individuals without disabilities [14]. The incidence of victimization is high among those with ID; the types of victimization include assault, exclusionary behavior, threats, verbal bullying, and sexual harassment [15,16,17]. Victimization is most frequently reported in school, group home, supported employment, family home, and community settings [18]. Studies have also revealed that victimization experiences are associated with poor mental health among individuals with ID [15,19]. Notably, polyvictimization is associated with the development of considerably more psychiatric symptoms relative to single repeated victimization, and it can lead to mental illness [20,21].

The caregiving provided to individuals with an ID is multifaceted and long term [22]. Research indicates that gender inequalities, old age, lack of companionship, and low socioeconomic status increase the risk of requiring long-term care [23,24]. Common challenges in long-term care include physical function, cognition, mental health, associated harms (e.g., accidents, abuse, and neglect), peer victimization (PV), implementation of acute care, mortality, and expenditures [25,26]. These challenges require urgent attention because long-term care remains the dominant form of ID services in Taiwan [27]; furthermore, the disability severity of most individuals in ID services ranges from moderate to profound (80%) [28]. Studies have indicated that almost half of all individuals in ID services have multiple disabilities, and approximately 60% have other impairments [29]. The most common disorders are epilepsy, cerebral palsy, and psychosis [30]. The combination of the aforementioned factors contributes to the physical and psychological complexity of long-term care. In Taiwan, psychiatric monitoring scales are rarely used among adults with ID in a long-term care setting.

Several scales can be used to assess individuals with mild-to-moderate ID; they include the Assessment of Dual Diagnosis, Reiss Screen for Maladaptive Behavior, and Mini Psychiatric Assessment Schedule for Adults with a Developmental Disability [31,32,33,34]. For individuals with severe-to-profound ID, the Diagnostic Assessment for the Severely Handicapped is a useful scale. [35]. The Psychopathology Inventory for Mentally Retarded Adults (PIMRA) can evaluate individuals with mild-to-profound ID [36]. Thus, the present study applied the PIMRA, which can assess the IDs of all levels of severity and is widely used internationally, to explore the distribution of psychiatric symptoms among adults with ID in Taiwan [37,38,39,40].

Most related studies have focused on adolescents’ victimization experience and mental health; however, few studies have explored the relationship between PV experience and mental health among adults with IDs. And because psychiatric symptoms are prone to periodic relapses, particularly among individuals with IDs, a longitudinal design was expressed in this research’s objectives [41]. The aim of this study is to investigate the various types of PV experienced by adults with ID, and the influence of PV on the long-term mental health status of adults with ID who receive long-term care. The hypotheses in this study are as follows: (a) different types of peer victimizations are associated with adults with ID’s psychiatric symptoms, and (b) the PV experience has a significant influence on mental health among adult users of ID services.

## 2. Materials and Methods

### 2.1. Study Design and Participants

A longitudinal follow-up design was adopted, and four surveys separated by 3-month intervals were conducted; demographic variables were recorded during the first survey. Major variables such as PV experience and psychiatric symptoms were recorded during each wave. The selected sample in the study was designed to have a representative coverage of regional urban, suburban, and rural areas spread across different service types. The domestic disability service type includes three forms of day service, night service, and 24 h accommodation and with the users of 4218, 184, and 13,058, respectively [3]. The present samples compose of stratified samples with a compatible proportion of 72, 11, and 93 in the three service types. The research protocol was approved by the Institutional Review Board of the Tri-Service General Hospital, National Defense Medical Center (1–105-05–173).

In the present study, enrolled users of ID services were older than 18 years of age and had official ID certificates. Individuals with ID were excluded if their moods could not be observed by their main caregiver. The questionnaires for an individual with ID were completed by that individual’s main caregiver in the ID services, who must have known that individual for at least 6 months. All caregivers had a background in psychology testing and were additionally trained in applying the questionnaires used in this study. Before the study began, the inter-rater reliability of the PIMRA scale was examined. To perform this, 40 participants’ data were collected and inter-rated. Each participant’s questionnaire was performed by two caregivers (raters) who were familiar with them and took turns caring for them. The inter-rater reliability result did not show disagreements in the McNemar’s test. After confirmation of the reliability, 219 adults with IDs were invited in the proceeding study; parental consent was not provided for 36 participants, and seven more adults were excluded because of their illogical responses in the psychiatric symptom questionnaires. Data collection was completed in 2020.

These 8 community-based long-term care ID services provide care to individuals with ID who require rehabilitation assistance pertaining to their basic independent living skills; they also provide services relating to daily operations, maintenance of physical functions, job training in the daytime, as well as respite, half-day, or full-day care services. The ID severity of 95% of the participants ranged from moderate to profound. Male participants accounted for approximately 55% of the sample. The average period of using long-term care ID services among the participants was 9 years. The participants were between 19 and 69 years old, and most (84%) of them were aged between 19 and 59 years. The services that the participants used generally had limited availability in Taiwan, where approximately only 10% of individuals with IDs are served by 266 disability services.

### 2.2. Assessment of Victimization Experience among Peers

Victimization experience among peers was assessed at baseline and 3, 6, and 9 months after the baseline assessment (i.e., four waves). The number of PV experiences reported at each time point were recorded and classified by frequency (i.e., never, once (1 incident), repeated (2–4 incidents)) during the study period. The types of PV examined in this research included physical force, exclusionary behavior, threats, verbal victimization, and sexual harassment [15,42]. The scale of PV experience was adopted from the Chinese version of the School Bullying Experience Questionnaire, with Cronbach’s Alpha value of 0.727 [43]. There was a significant correlation between peer victimization and mental health, which showed good prediction on the validity [15,44]. To assess physical force, the following question was asked: have you been subjected to physical force? For the other four types of PV, similar questions were also asked. Each item was answered by the main caregiver and evaluated on a 5-point scale (0 = *never*, 1 = *1–2 times per wave*, 2 = 2–3 *times per month*, 3 = *2–3 times per week*, and 4 = *1 time per day*) to measure PV frequency. Thereafter, the four waves of PV frequency data were summed and classified by frequency (never, once (PV frequency code = 1), and repeated PV) during the study period. Furthermore, participants who experienced more than two types of PV in a single wave were defined as having polyvictimization experiences. The number of polyvictimization experiences for each wave was recorded and classified by number of types of PV (i.e., zero to more than three types).

### 2.3. Assessment of Psychiatric Symptoms

The psychiatric symptoms of adults with ID were measured using the PIMRA [36]. In practice, the PIMRA can be used to screen, assess, and monitor mild to profound ID as well as to guide referrals [36]. The PIMRA exhibits high reliability and validity, with an internal consistency of 0.85, split-half reliability of 0.88, and construct validity 0.87; it is also a robust measurement tool for assessing the psychopathological status of individuals with ID [36,40,45]. The Taiwanese version of the PIMRA was developed on the basis of the Diagnostic and Statistical Manual of Mental Disorders (*DSM*) *III* and *IV*. The test–retest reliability of the Taiwanese PIMRA was assessed in a 2-week preliminary study involving 40 participants; the results revealed an intraclass correlation coefficient of 0.95 and internal consistency α of 0.89. The McNemar’s test was used on the inter-rater reliability and did not show disagreement on each dimension. The average agreement rate on each psychopathology dimension was 77–98% between 2 caregivers. And the instrument’s validity was verified by experts in psychiatry, medical sociology, and healthcare management respectively. Furthermore, in countries such as the United Kingdom, Norway, the Netherlands, and Italy, the PIMRA has been translated into other languages for international use [37,38,39,40].

The PIMRA comprises seven subscales of psychopathology (schizophrenia, affective disorder, psychosexual disorder, adjustment disorder, anxiety disorder, somatoform disorder, and personality disorder) and an eighth subscale for measuring inappropriate mental adjustment. Each subscale has seven items (1 point per item, with 4 or more points on a psychopathology symptom subscale indicating the presence of the psychopathology symptom). In the present study, a participant who exhibited any of the seven aforementioned psychopathology symptoms was defined as having psychiatric symptoms. Moreover, psychiatric symptoms and their period prevalence were tracked for the four waves.

### 2.4. Measurement of Covariates

The variables of the characteristics associated with adult psychiatric symptoms were recorded and used as covariates for controlling potential confounding effects. Studies have indicated that being female, being poor, having a severe ID, having 24 h accommodation agency services, having divorced parents, and having PV experiences are risk factors for mental health problems among individuals with ID [15,46,47].

The aforementioned risk factors for adult psychiatric symptoms were examined and analyzed in the present study. The demographic variables were stratified by sex, age, education level, ID severity, and secondary disability diagnosis.

The ID services-related variables comprised the type of ID service received, interest in course, skills learned, regular medication, activities of daily living (ADL), and PV experience.

### 2.5. Statistical Analysis

The questionnaires were each checked carefully when returned, and proactive communication with the ID services was maintained to complete any missing data. To ensure data quality and to detect outliers, continuous variables such as age were checked by Z values (|3.29|) [48]. Additionally, categorical variables were checked by frequency in SPSS statistical software. The seven cases found of illogical responses with conflict among the PIMRA subscales were excluded and the included data checked were acceptable without obvious outliers. A chi-square test was performed to compare the categorical variables of participants with psychiatric symptoms and those without psychiatric symptoms. For psychiatric symptom outcomes, logistic regression was performed to analyze the independent predictive variables of personal characteristics, PV experience, PV type, and polyvictimization. In the logistic regression, PV experience frequency was analyzed using the first model, five types of PV were dichotomized as “0” (never) and “ever” (1–4) using the second model, and the polyvictimization was analyzed using the third model. For all tests conducted in the present study, statistical significance was set at *p* < 0.05, and analyses were conducted using IBM SPSS Statistics V.22.0 (IBM, Armonk, NY, USA).

## 3. Results

### 3.1. Participant Characteristics

The sample of the present study comprised 176 adults with ID (98 male and 78 female adults). The characteristics of the participants are summarized in Table 1. Among the participants, 36.4% were aged 18–30 years, 18.8% were aged 31–40 years, 15.3% were aged 41–50 years, and 29.5% were aged ≥51 years. Approximately 52% of the participants had more than 9 years of education. Most of the participants had moderate (37%) or severe (36%) ID, whereas 21% and 5% had profound and mild ID, respectively. Approximately 46% of the participants were diagnosed as having a secondary disability.

Most participants were served by 24 h accommodation agencies (52%) followed by day service agencies (40%) and night service agencies (6%). Regarding the training classes provided by the ID services, 93% of the participants indicated that they were interested in these classes; the largest proportion of participants (43%) received basic skills training, and 57% received training for one or more skills. With respect to regular medication, 34% of the participants took medications for physical conditions, and 33% of people took medications for both physical and psychiatric conditions (i.e., psychotropics). In terms of ADL dependence, most of the adults were dependent (34.7%) followed by moderately dependent (33.5%), heavily dependent (10.8%), mildly dependent (10.8%), and independent (10.2%).

### 3.2. Prevalence of Psychiatric Symptoms

The present study measured the prevalence of psychiatric symptoms among the participants (*n* = 176) in the four waves by using the PIMRA (Table 2). In terms of the participants’ average prevalence as obtained from four measurements, 14.3% had at least one PIMRA psychiatric symptom with a cumulative period prevalence of 31.8%. Among the participants, the average prevalence of schizophrenia was 3.4% with a period prevalence of 9.1% (*n* = 16); the average prevalence of affective disorder symptoms was 1.6% with a period prevalence of 4.5% (*n* = 8); the average prevalence of psychosexual disorder was 0.1% with a period prevalence of 0.6% (*n* = 1); the average prevalence of adjustment disorder was 5.3% with a period prevalence of 15.3% (*n* = 27); the average prevalence of anxiety disorder was 5.7% with a period prevalence of 16.5% (*n* = 29); the average prevalence of somatoform disorder was 5.8% with a period prevalence of 13.6% (*n* = 24); the average prevalence of personality disorder was 3.0% with a period prevalence was 8.5% (*n* = 15).

### 3.3. PV Experience

The PV experience of the participants was assessed four times at 3-month intervals. Approximately 40% experienced PV (including physical force, exclusionary behavior, threats, verbal victimization, and sexual harassment) at least once during the study period.

The four-wave measurements revealed that 10%–16% of the participants experienced PV involving physical force with a cumulative prevalence of up to 26% during the study period; PV involving exclusionary behavior, threats, verbal victimization, and sexual harassment affected 6–10%, 2–7%, 9–15%, and 3–5% of the participants, respectively. Moreover, among the participants, the cumulative prevalence of PV involving exclusionary behavior, threats, verbal victimization, and sexual harassment reached 18%, 10%, 22%, and 7%, respectively, during the study period. Approximately 22% of all the participants experienced PV characterized by polyvictimization (Table 3).

Among the participants with psychiatric symptoms, approximately 45% experienced physical force, approximately 40% experienced exclusionary behavior, almost 20% received threats, approximately 46% experienced verbal victimization, almost 14% experienced PV involving sexual harassment, and 50% experienced polyvictimization; all these figures were significantly higher than those reported by the group without psychiatric symptoms.

### 3.4. Factors Associated with Psychiatric Symptoms among Participants

The results presented in Table 1 and Table 3 suggested that independent factors such as secondary disability diagnosis, medication use, PV experience frequency, PV type, and polyvictimization were significantly associated with psychiatric symptoms. The relevant factors associated with psychiatric symptoms among the participants were analyzed using a logistic regression model (Table 4). Among the personal characteristics, sex, education, training skills, and ID severity were not associated with psychiatric symptoms. Notably, participants aged between 18 and 30 years (odds ratio (OR) = 4.88), 31 and 40 years (OR = 4.83), and 41 and 50 years (OR = 5.76) exhibited a significantly higher risk for psychiatric symptoms relative to participants aged above 50 years, indicating that psychiatric risk is higher among adults with ID who were aged less than 50 years. Having a secondary disability diagnosis was associated with an increased risk of developing psychiatric symptoms (OR = 2.54). Furthermore, the use of medication for both physical and psychiatric conditions (i.e., psychotropics) was associated with having psychiatric symptoms (OR = 3.94) (Table 4).

In Model 1, after adjustments were made for confounding factors, participants who experienced repeated PV (2–4 times) exhibited a significantly higher OR (13.55, 4.71–38.95) relative to those who did not experience PV. In Model 2, after adjustments were made for confounding factors, PV involving physical force was a significant risk factor for psychiatric symptoms with an OR of 3.31 (1.13–9.70), and PV involving verbal victimization was a significant risk factor for psychiatric symptoms with an OR of 8.36 (1.65–42.46). In Model 3, after adjustments were made for confounding factors, polyvictimization was a significant risk factor for psychiatric symptoms with an OR of 15.16 (5.04–45.67) (Table 4).

## 4. Discussion

In the present study, an average of 14% of the participants exhibited psychiatric symptoms, as measured by the PIMRA, at each measurement time point (separated by 3-month intervals), and 32% exhibited psychiatric symptoms during the 1-year study period. Among the participants, the prevalence of at least one PIMRA psychiatric symptom was 32% and 18% of the participants exhibited at least two PIMRA psychiatric symptoms during the study period. The average prevalence of psychiatric symptoms during the 1-year period (14%) is similar to the 12.1% reported in Taiwan’s National Disability Registration Database [49].

For average prevalence, the three leading PIMRA psychiatric symptoms during the study period were adjustment disorder (5%), anxiety disorder (6%), and somatoform disorder (6%), whereas psychosexual disorder (0.1%) had the lowest average prevalence. During the study period, the overall prevalence of adjustment disorder, anxiety disorder, and somatoform disorder was 15%, 16%, and 14%, respectively, while psychosexual disorder cumulated at 0.6%. Relative to other studies, the figures relating to PIMRA psychiatric symptoms are lower in the present study. In the United States, a study that used the PIMRA reported that anxiety disorder had the highest prevalence at 23% [50]; in Sweden, a study revealed that anxiety disorder (26%) and adjustment disorder (21.1%) had the highest prevalence [12]. In both PIMRA-based and non-PIMRA-based studies that have investigated individuals with ID, the prevalence of adjustment disorder, anxiety disorder, and somatoform disorder ranged from 0.5% to 21.1%, <2% to 26%, and 3.1% to 24.4%, respectively [12,51,52,53]. With the PIMRA instrument, our findings showed compatibility with previous literature stating that adjustment disorder, anxiety disorder, and somatoform disorder are the most prevalent symptoms and psychosexual disorder was rarely reported among individuals with ID [12,51,52,53,54]. Thus, the seven subscales in PIMRA instruments could be efficiently adopted to monitor the individual with psychiatric disorders. The minor differences between the findings of the present study and other studies could be attributed to the sample used in the present study; that is, the present study only examined participants from ID services, which might have provided superior management of these individuals’ conditions. Although the prevalence of the three aforementioned disorders varied significantly among multiple studies, they were invariably the three most common psychiatric symptoms exhibited by individuals with ID.

Individuals with ID who have adjustment disorder, anxiety disorder, or somatoform disorder encounter more difficulties in their daily lives. Research has indicated that adjustment disorder is a precursor of anxiety and depressive disorders [55]; individuals with anxiety disorder frequently exhibit negative cognitions, which have negative effects on their daily living functions and are a risk factor for challenging behaviors [56,57,58]; individuals with somatoform disorder exhibit substantially greater functional disability and role impairment [59]. Moreover, anxiety and somatoform disorders can lead to suicidal ideation and attempts in the general population [60,61]. These mentioned psychiatric symptoms increase the difficulty of providing adequate care in ID services. Therefore, ID service caregivers must pay extra attention to individuals who exhibit the three aforementioned psychiatric symptoms.

Furthermore, the prevalence of psychiatric symptoms fluctuated during the study period, indicating that psychiatric symptoms tend to relapse frequently [62,63]. Studies have revealed that awareness of changes in daily life and removal of stressors can ameliorate the signs and symptoms of psychiatric disorders [55,62,64]; for example, a short-term exercise program can reduce anxiety states [56]. In addition, mental health should be maintained by improving adherence to psychiatric medication [65] and creating stable environments [66]. Therefore, the psychiatric symptoms of individuals with ID tend to fluctuate and require continual and focused care.

In the present study, the participants who experienced PV exhibited more psychiatric symptoms than those who did not experience PV. Among the participants, 39.8% experienced PV and 25.6% experienced repeated PV during the study period. Notably, in the present study, the reported occurrence of PV is lower relative to the figures reported in other studies. A study conducted in Spain reported a 96.9% lifetime prevalence of victimization among adults with ID who received services from social initiative entities [16]. An Australia-based study discovered that most adults with ID experienced abuse (emotional abuse, 83.3%; sexual abuse, 68.8%; physical abuse, 68.3%) in their childhood institutions [67]. A Taiwan-based study reported that 70% of adolescents with ID experienced PV [15]. In the present study, the PIMRA was used to evaluate the mental status of participants, thereby enabling relevant personnel to develop appropriate steps for implementing improvements.

Studies have indicated that PV experience is associated with mental illness [21,68]. A study reported that PV is related to mental illness in both individuals with and without disabilities [69]. The present study revealed that PV experience is a key risk factor for psychiatric symptoms among adult users of ID services. Notably, adults with IDs who experienced repeated PV (2–4 times; 25.6%) during the study period exhibited a 13-fold higher risk of psychiatric symptoms than those who did not have such experiences. Adults with ID who experienced polyvictimization (21.6%) exhibited a 15-fold higher risk of psychiatric symptoms compared with those who did not have such experiences during the study period. Thus, the association of psychiatric symptoms with polyvictimization is greater than that with single repeated victimization [20]. Among the various types of PV, physical force (25.6%) and verbal victimization (21.6%) were, respectively, associated with a 3.3- and 8.3-fold higher risk of PIMRA psychiatric symptoms. Studies have revealed that among individuals with ID, physical force (68%) and verbal victimization (64%) are the most common types of PV and risk factors for psychiatric symptoms [15,67,70]. Factors including age, types of caregiving, psychotropic use, and psychiatric symptoms were associated with PV experience in the present study (data were not shown). Research has shown similarly that being alone, being younger, being physically weaker, having secondary disabilities, and having a more severe disability would make victimization more likely [18]. Individuals with ID who experienced victimizationrelated emotional problems, low self-esteem, and a negative social impact [71]. Furthermore, in the aspect of interpersonal relations, victims are prone to become perpetrators, resulting in more victims [25]. In response to the occurrence of victimization, although individuals had learned how to recognize, avoid, and report victimization, they still needed to receive psychosocial intervention and practice self-advocacy skills to resolve victimization [72,73]. Researchers have advocated for the provision of effective support (e.g., an individual who can speak and establish trust with a victim, protect the victim against victimization, provide support when victimization occurs, and respond to an individual’s reports of victimization) [18,74].

Among the participants of the present study, the prevalence of psychotropic use among regular medication users was 33% at baseline. The psychiatric medication prevalence reported in the present study is low relative to other studies (37–58%) [75,76]. Moreover, the results indicated that adults with ID who took psychotropics regularly exhibited a 3.94-fold higher risk of psychiatric symptoms. The psychiatric symptoms of adults with ID who took psychotropics were regarded as being under control (of the 56 participants who exhibited PIMRA psychiatric symptoms, 32 were taking psychotropics). Nevertheless, they were still at a higher risk of PIMRA psychiatric symptoms in the 1-year follow-up. Although the aforementioned participants used psychotropics, their mental health must still be monitored; therefore, future studies should explore the efficacy of medications for managing psychiatric symptoms in those with ID.

Individuals with secondary disabilities are a high-risk group for psychopathy [77]. In the present study, approximately 45% of participants in ID services were diagnosed with secondary disabilities; this finding corresponds to the 45–66.9% prevalence reported in other studies [16,78]. Our results also revealed that individuals with secondary disabilities exhibited a 2.5-fold higher risk of PIMRA psychiatric symptoms relative to those without secondary disabilities. Therefore, caregivers should pay more attention to the mental health of adults with ID who have secondary disabilities.

Among the ID services, studies have indicated that small-scale community arrangements (similar to day service agencies in the present study) and semi-independent living (similar to night service agencies in the present research) lead to more community involvement relative to 24 h accommodation agencies [79,80]. In our analysis, adults with ID who used 24 h accommodation agencies exhibited a 3-fold higher risk of psychiatric symptoms relative to those who used day or night service agencies. Inadequate social and environmental support are risk factors for poor mental health among individuals with ID [70,81]. Individuals with ID who used 24 h accommodation agencies exhibited poorer health (including infectious diseases, skin diseases, and psychiatric disorders) than non-24 h accommodation agencies [82]. Caregivers and relevant units could provide increased social participation and environmental support to help improve the mental health of such individuals.

## 5. Conclusions

In conclusion, PV, younger age, regular medication use, secondary disability, and use of ID services were associated with psychiatric symptoms among adults with ID. The PIMRA used in the present study is an effective monitoring tool for ID services caregivers. The empirical observation results obtained through the PIMRA during the 1-year study period revealed a significant correlation between the psychiatric symptoms and PV and demonstrated the practicality of the PIMRA as a mental health tool. Thus, the PIMRA can be used as a practical tool in ID services to improve the mental health of adults with IDs. The strengths of this study are as follows: (1) the PIMRA can be used as a practical tool in ID services to improve the mental health of adults with ID; (2) in contrast to other ID victimization studies, where the perpetrators are mostly caregivers or family members, the present study explored victimization between peers (i.e., perpetrators are adults with ID). The weakness of this research is that the eight ID services provided excellent caregiving and, therefore, this may lead to an underestimation of PV experiences and the psychological symptoms of individuals with IDs. In summary, caregivers and parents could pay more attention to PV occurrence among adults with ID, and learn more listening, communication and psychosocial intervention skills; as previously described, this will help social adjustment for those who are victimized. The interpretation of the results and promotion of the PIMRA can help improve the mental health of adults with ID who are receiving ID services, and the PIMRA scale can be adopted as an evaluation tool for a mental health intervention program in future research.

## Figures and Tables

**Table 1:** Characteristics of adults with intellectual disabilities (*n* = 176).

	PIMRA Psychiatric Symptoms		
Characteristics	No (*n* = 120) *n* (%)	Yes (*n* = 56) *n* (%)	Total (*n* = 176) *n* (%)
Sex			
Male	64 (53.3)	34 (60.7)	98 (55.7)
Female	56 (46.7)	22 (39.3)	78 (44.3)
Age			
18–30	43 (35.8)	21 (37.5)	64 (36.4)
31–40	21 (17.5)	12 (21.4)	33 (18.8)
41–50	14 (11.7)	13 (23.2)	27 (15.3)
51+	42 (35.0)	10 (17.9)	52 (29.5)
Education			
≤9 grades	59 (49.2)	24 (42.9)	83 (47.2)
>9 grades	61 (50.8)	32 (57.1)	93 (52.8)
Degree of ID			
Mild	9 (7.5)	0	9 (5.1)
Moderate	45 (37.5)	21 (37.5)	66 (37.5)
Severe	40 (33.3)	24 (42.9)	64 (36.4)
Profound	26 (21.7)	11 (19.6)	37 (21.0)
2nd disability diagnosis ^a^			
Yes	48 (40.0)	32 (57.1) *	80 (45.5)
No	72 (60.0)	24 (42.9)	96 (54.5)
Type of ID services			
Day service agencies	54 (45.0)	18 (32.1)	72 (40.9)
Night service agencies	7 (5.8)	4 (7.1)	11 (6.3)
24 h accommodation agencies	59 (49.2)	34 (60.7)	93 (52.8)
Interested in course			
All	17 (14.2)	8 (14.3)	25 (14.2)
Most	46 (38.3)	18 (32.1)	64 (36.4)
Few	50 (41.7)	25 (44.6)	75 (42.6)
None	7 (5.8)	5 (8.9)	12 (6.8)
Skills learned			
Basic ability	55 (45.8)	20 (35.7)	75 (42.6)
1 skill	39 (32.5)	23 (41.1)	62 (35.2)
2 skills	22 (18.3)	11 (19.6)	33 (18.8)
≥3 skills	4 (3.3)	2 (3.6)	6 (3.4)
Regular medication			
None	45 (37.5)	13 (23.2) ***	58 (33.0)
Physical	49 (40.8)	11 (19.6)	60 (34.1)
Psychiatric	—	—	—
Both physical and psychiatric	26 (21.7)	32 (57.1)	58 (33.0)
ADL ^b^			
Independent 100	14 (11.7)	4 (7.1)	18 (10.2)
Mildly dependent 91–99	15 (12.5)	4 (7.1)	19 (10.8)
Moderately dependent 61–90	34 (28.3)	25 (44.6)	59 (33.5)
Heavily dependent 21–60	11 (9.2)	8 (14.3)	19 (10.8)
Dependent 0–20	46 (38.3)	15 (26.8)	61 (34.7)

a. Another diagnosed disability coexisting with the main intellectual disability. b. ADL: activities of daily living. * *p*-value < 0.05, *** *p*-value < 0.001.

**Table 2:** Four-wave prevalence and period prevalence of PIMRA psychiatric symptoms (*n* = 176).

PIMRA	Average Prevalence *n* (%)	Period Prevalence *n* (%)
Schizophrenia	6.0 (3.4%)	16 (9.1%)
Affective disorder	2.8 (1.6%)	8 (4.5%)
Psychosexual disorder	0.3 (0.1%)	1 (0.6%)
Adjustment disorder	9.3 (5.3%)	27 (15.3%)
Anxiety disorder	10.0 (5.7%)	29 (16.5%)
Somatoform disorder	10.3 (5.8%)	24 (13.6%)
Personality disorder	5.3 (3.0%)	15 (8.5%)
Any symptom	25.3 (14.3%)	56 (31.8%)

**Table 3:** Peer victimization experience during 1-year study period.

PIMRA Psychiatric Symptoms			
Characteristics	No (*n* = 120) *n* (%)	Yes (*n* = 56) *n* (%)	Total (*n* = 176) *n* (%)
PV experience frequency ^c^			
0	89 (74.2)	17 (30.4) ***	106 (60.2)
Once (1 time)	16 (13.3)	9 (16.1)	25 (14.2)
Repeated (2–4)	15 (12.5)	30 (53.6)	45 (25.6)
*PV types* ^d^			
Physical force ^c^			
0	100 (83.3)	31 (55.4) ***	131 (74.4)
Once (1 time)	14 (11.7)	10 (17.9)	24 (13.6)
Repeated (2–4)	6 (5.0)	15 (26.8)	21 (11.9)
Exclusionary behavior ^c^			
0	111 (92.5)	34 (60.7) ***	145 (82.4)
Once (1 time)	6 (5.0)	9 (16.1)	15 (8.5)
Repeated (2–4)	3 (2.5)	13 (23.2)	16 (9.1)
Threats ^c^			
0	113 (94.2)	45 (80.4) **	158 (89.8)
Once (1 time)	7 (5.8)	6 (10.7)	13 (7.4)
Repeated (2–4)	0	5 (8.9)	5 (2.8)
Verbal victimization ^c^			
0	108 (90.0)	30 (53.6) ***	138 (78.4)
Once (1 time)	3 (2.5)	6 (10.7)	9 (5.1)
Repeated (2–4)	9 (7.5)	20 (35.7)	29 (16.5)
Sexual harassment ^c^			
0	115 (95.8)	48 (85.7) *	163 (92.6)
Once (1 time)	2 (1.7)	2 (3.6)	4 (2.3)
Repeated (2–4)	3 (2.5)	6 (10.7)	9 (5.1)
Polyvictimization ^c^			
0	89 (74.2)	17 (30.4) ***	106 (60.2)
1 type	21 (17.5)	11 (19.6)	32 (18.2)
2 types	4 (3.3)	15 (26.8)	19 (10.8)
≥3 types	6 (5.0)	13 (23.2)	19 (10.8)

c. Period prevalence of peer victimization experience based on four-wave data. d. PV types: physical force, exclusionary behavior, threats, verbal victimization, and sexual harassment. * *p*-value < 0.05, ** *p*-value < 0.01, *** *p*-value < 0.001.

**Table 4:** Logistic regression analysis of characteristics associated with PIMRA psychiatric symptoms.

Variables	Model 1	Model 2	Model 3
	OR (95% CI)	OR (95% CI)	OR (95% CI)
Age			
18–30	4.05 (0.90–18.23)	4.65 (0.99–21.80)	4.88 (1.13–21.14) *
31–40	5.21 (1.25–21.76) *	5.19 (1.14–23.68) *	4.83 (1.16–20.10) *
41–50	6.08 (1.39–26.61) *	6.81 (1.51–30.64) *	5.76 (1.25–26.55) *
50+	ref	ref	ref
2nd disability diagnosis ^a^			
Yes	2.54 (1.01–6.36) *	3.03 (1.16–7.96) *	2.75 (1.09–6.98) *
No	ref	ref	ref
Type of ID services			
24 h accommodation agencies	2.80 (0.90–8.70)	2.80 (0.86–9.11)	3.32 (1.07–10.23) *
Day/night service agencies	ref	ref	ref
Regular medication			
Physical	0.66 (0.22–2.06)	0.71 (0.22–2.26)	0.73 (0.24–2.28)
Psychiatric	—	—	—
Both physical and psychiatric	3.94 (1.41–10.98) **	3.56 (1.24–10.20) *	4.07 (1.44–11.48) **
None	ref	ref	ref
PV experience frequency ^c^			
Repeated (2–4 times)	13.55 (4.71–38.95) ***	—	—
Once (1 time)	3.02 (0.93–9.78)	—	—
0	ref	ref	ref
*PV types* ^d^			
Physical force ^c^			
Ever	—	3.31 (1.13–9.70) *	—
0	—	ref	—
Exclusionary behavior ^c^			
Ever	—	2.03 (0.36–11.50)	—
0	—	ref	—
Threats ^c^			
Ever	—	0.23 (0.04–1.36)	—
0	—	ref	—
Verbal victimization ^c^			
Ever	—	8.36 (1.65–42.46) *	—
0	—	ref	—
Sexual harassment ^c^			
Ever	—	2.19 (0.39–12.27)	—
0	—	ref	—
Polyvictimization ^c^			
Yes	—	—	15.16 (5.04–45.67) ***
No	—	—	ref

a. Another diagnosed disability coexisting with the main intellectual disability. c. Period prevalence of peer victimization experience based on four-wave data. d. PV types: physical force, exclusionary behavior, threats, verbal victimization, and sexual harassment. * *p*-value < 0.05, ** *p*-value < 0.01, *** *p*-value < 0.001.

## Data Availability

The data presented in this study are available on request from the corresponding author. The data are not publicly available due to privacy.
